# Assessment of cervical lymph node metastasis for therapeutic decision-making in squamous cell carcinoma of buccal mucosa: a prospective clinical analysis

**DOI:** 10.1186/1477-7819-10-253

**Published:** 2012-11-22

**Authors:** Harald Essig, Riaz Warraich, Gulraiz Zulfiqar, Madiha Rana, André Michael Eckardt, Nils-Claudius Gellrich, Majeed Rana

**Affiliations:** 1 Department of Cranio-Maxillofacial Surgery, Hannover Medical School, Carl-Neuberg-Street 1, Hannover, D-30625, Germany; 2 Department of Oral and Maxillofacial Surgery, King Edward Medical University, Lahore, Pakistan

**Keywords:** Squamous cell carcinoma, Prognosis, Oral cavity, Buccal mucosa, Lymph node metastasis

## Abstract

**Background:**

Cervical metastasis has a tremendous impact on the prognosis in patients with carcinomas of the head and neck and the frequency of such spread is greater than 20% for most squamous cell carcinomas. With emerging evidence, focus is shifting to conservative neck procedures aimed at achieving good shoulder function without compromising oncologic safety. The purpose of this study was to analyze the pattern of nodal metastasis in patients presenting with squamous cell carcinoma of buccal mucosa.

**Materials and methods:**

This was a prospective clinical analysis of patients who were histologically diagnosed with squamous cell carcinoma of the buccal cavity and clinically N1 and had not received treatment anywhere else. Patients were analyzed for age and sex distribution, tumor staging, location, and metastasis.

**Results:**

The incidence of metastatic lymph node in T4 (*n*=44) was the highest, that is, level I was 100% (44/44), level II was 43.18% (19/44), level III was 15.90% (7/44), and level IV was 4.5% (2/44). Level V was free of metastasis. Among T3 (*n*=10) lesions, incidence of metastasis in level I was 100% (10/10), level II was 20% (2/10), and level III, IV, and V were free of metastasis. Among T2 (*n*=6) lesions, incidence of lymph node metastasis in level I was 100% (6/6) and all other levels of lymph nodes were found free of metastasis.

**Conclusion:**

Lymphatic spread from carcinoma of the buccal mucosa is low. Involvement of level IV is seen in only 3% of patients. A more conservative approach to the neck in patients with carcinoma of the buccal mucosa is recommended.

## Background

Squamous cell carcinoma (SCC) in the head and neck region occurs primarily in the oral cavity and oropharynx and is generally regarded as a disease of the elderly [1]. In contrast to other sites of oral cancer, the incidence of the buccal carcinoma is increasing, especially in younger age groups [2,3]. The incidence of buccal carcinoma is much higher in Asia. In Southeast Asia, the disease is the most common form of oral cavity cancer. The higher rate of buccal carcinoma is likely related to the widespread practice of betel nut chewing. Betel nut, composed mainly of the fruit of the Areca Palm and often mixed with tobacco, is placed along the buccal mucosa to induce a feeling of euphoria. The anatomical and physiological milking muscle action predispose to an early invasion and metastasis of buccal carcinoma [4]. Multimodal management of buccal carcinoma include surgery, radiotherapy, chemotherapy, and the combination of the above three. It depends upon tumor factors such as site, size (T stage), location, multiplicity, proximity to bone, pathological features, histology grade, depth of invasion, and status of cervical lymph nodes. The patient factors include previous treatments and medical condition of the patient. Competence and convenience of the surgeon, economics and compliance of the patients, and expected complications also play their role in treatment planning [5]. Approximately 300 lymph nodes are located in the head and neck and they comprise 30% of the total 800 lymph nodes in the human body. Cervical metastasis has a tremendous impact on the prognosis in patients with carcinomas of the head and neck and the frequency of such spread is greater than 20% for most squamous cell carcinomas [6]. Predictive factors of cervical metastasis are primary site, primary tumor size, degree of differentiation of tumor, perineural invasion, perivascular invasion, inflammatory response, and tumor DNA content (ploidy) [7]. It is described that 49% occult metastasis in cervical lymph nodes in patients presenting with squamous cell carcinoma of buccal mucosa [8]. Level I was the most common site for nodal metastases (100%), followed by level II (32%), level III (16%), and level IV (8%) [9]. Despite the development of multimodal treatment options, the prognosis remains relatively poor. After tongue carcinoma, the manifestation of occult lymph node metastasis of buccal carcinoma is observed more often than in any other cancer of the oral cavity [10]. An overall 5-year survival rate of 65% is described in the literature, although the distribution of tumor stages was about the same compared to the preceding 10-year period [11,12]. Better survival was related to a more aggressive treatment of the neck even in early tumor stages and to adjuvant radiotherapy in advanced tumor stages. A considerable number of patients had to be upstaged after elective neck dissection due to occult lymph node metastasis. The number of lymph node metastasis turned out to be of prognostic value. Only a few investigations have been done into the metastasis of squamous cell carcinoma of the buccal mucosa. But it is striking that the incidence of cervical lymph node metastasis from cancer of the buccal mucosa is significant.

The aim of this study is to analyze our data on the pattern of nodal metastasis in patients presenting with squamous cell carcinoma of buccal mucosa, their topographic distribution in different levels of cervical lymph nodes to provide grounds for an appropriate and optimal type of neck dissection required to obtain valid criteria for therapeutic decision-making in clinical routine.

## Materials and methods

The study was approved by the local ethics committee at the King Edward Medical University Lahore (KEMU-2008/602). Study subjects were enrolled in a clinical protocol reviewed and approved by the institutional cancer board. Before the beginning of the study, written informed consent was obtained from each patient.

### Patients

Patients were enrolled from the King Edward Medical University, Department of Oral and Maxillofacial Surgery. Sixty patients with tumor sizes T2 to T4 and clinically proven N1 neck were evaluated for the presence of metastasis in subsequent levels. All patients were provided with an informed consent and completed a detailed questionnaire, which included information on demographics, smoking and alcohol use, betel nut chewing, current medications, general health, and dental care history.

### Sample size

The calculated sample size was 60 cases with 95% confidence level, 12% margin of error, and taking expected percentage of metastasis in level III, that is, 32% in patients of squamous cell carcinoma of buccal mucosa undergoing extended supra-omohyoid neck dissection/selective (levels I to V).

### Study including criteria and protocol

Only patients aged 25 to 75 years with squamous cell carcinoma of buccal mucosa, tumor size T2 to T4 and clinically involved N1 were included in this study. Potential participants would have been excluded from the study because of history of being previously operated, systemic metastasis, already irradiated, co-morbid medical conditions resulting in fitness problem for general anesthesia. The clinical inclusion and exclusion criteria are shown in Table 1.

**Table 1:** Patient’s preoperative demographic data

**Inclusion criteria**	**Exclusion criteria**
Age 25 to 75 years, irrespective of gender	Previously operated
Histopathologically proven cases of squamous cell carcinoma of buccal mucosa	Systemic metastasis (detected by means of chest X-ray, abdominal ultrasound, and CT scan)
Primary tumor site in oral cavity is buccal mucosa	Unfit for general anesthesia
Clinical tumor size T2 to T4	Previously irradiated
Lymph node involvement: N1 (clinically and with sonographic examination)	Without written informed consent

All patients provided an informed consent and completed a detailed questionnaire, which included information on demographics, smoking and alcohol use, betel nut chewing, current medications, general health, and dental care history.

### Data collection

Incisional biopsy proven patients with tumor sizes T2 to T4, N1, M0 of oral squamous cell carcinoma of buccal mucosa presenting at the Department of Oral & Maxillofacial Surgery, King Edward Medical University, Lahore were admitted and prepared for surgery. Grading of the tumor among all patients was done according to the American Joint Committee on Cancer (AJCC)/UICC 2002 TNM classification. The demographic variables (that is, name, age, sex, address) will be recorded and informed consent will be taken.

**Figure 1:**
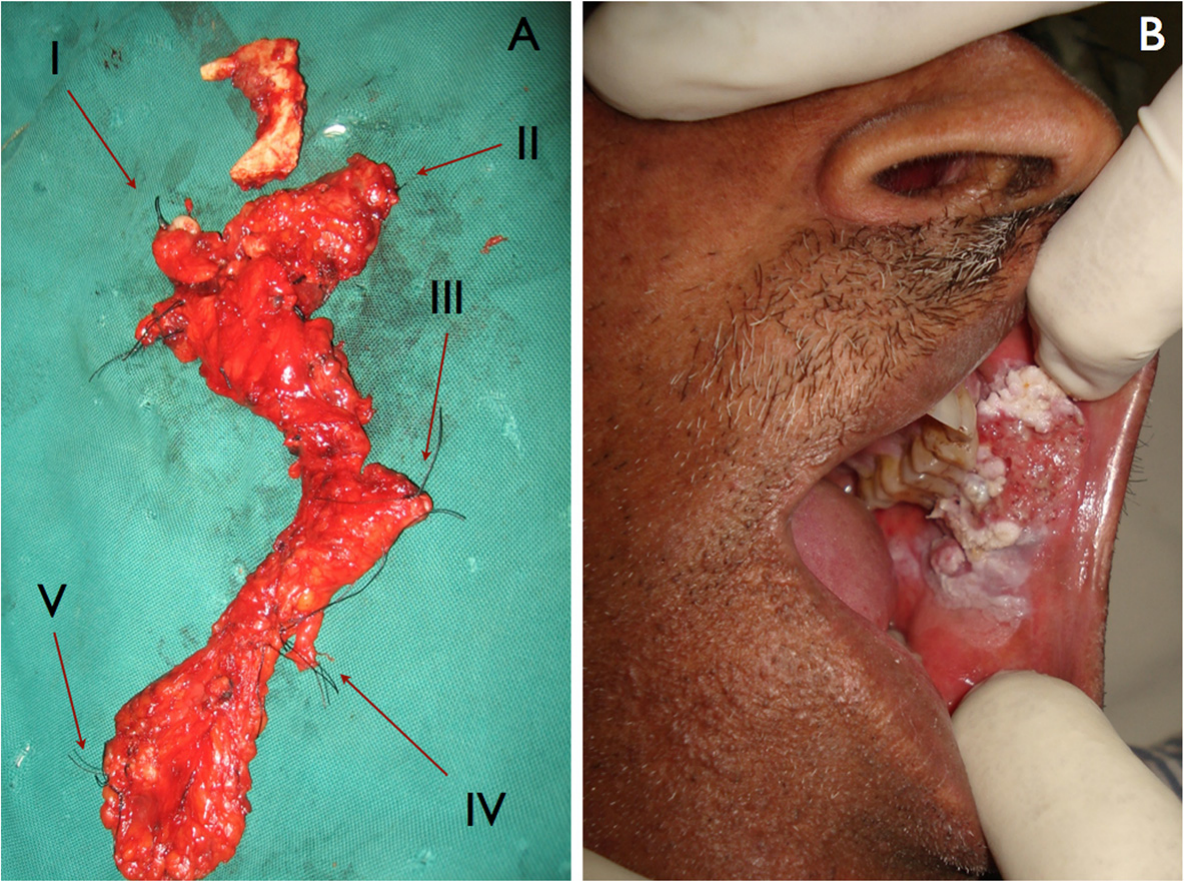
**(A) demonstrates the margins of all resected specimens of primary tumor and cervical lymph nodes.** Level I was marked with one silk string, level II with two silk strings, level III with three silk strings, level IV with four silk strings, and level V with five silk strings, (**B**) a 51-year-old patient with a squamous cell carcinoma of buccal mucosa and clinically proven N1 neck.

**Figure 2:**
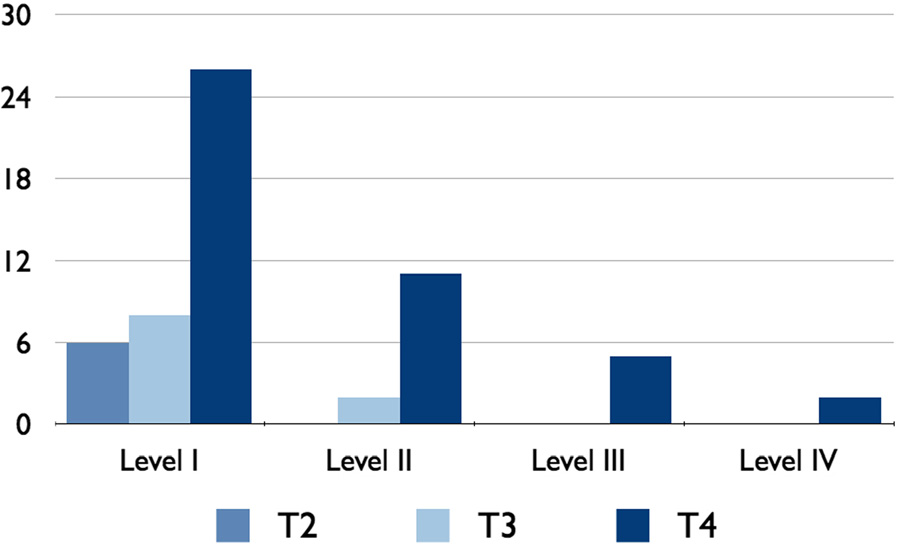
**Metastatic lymph node was the highest, that is, level I was 100% (44/44), level II was 43.18% (19/44), level III was 15.90% (7/44) and level IV was 3% (2/44).** Level V was free of metastasis.

Resection of the tumor with a safe margin of 1 cm to 1.5 cm was done in all patients. Moreover, neck dissection was done by one experienced operator in all patients and the extent of neck dissection was extended supra-omohyoid/selective (levels I to V). Margins of all resected specimens of primary tumor and cervical lymph nodes were marked with silk orientation sutures (Figure 1). Level 1 was marked with one silk string, level II with two silk strings, level III with three silk strings, level IV with four silk strings, and level V with five silk strings (Figure 2). The marked specimen along with biopsy form containing details of history and description of orientation silk strings was then sent for histopathological examination in which presence or absence of metastatic tumor cells was noted in level I to V lymph nodes. The above information was collected on performa attached as annexure-I.

### Statistical analysis

The collected information was transferred to SPSS (Statistical Package for the Social Sciences) version 11.5. The results will be presented in the form of frequencies and percentages. Qualitative variables such as levels of cervical lymph nodes (levels I, II and III, IV, V) and sex were presented as percentages and frequencies. Data were stratified for size of tumor (T2 to T4) to address effect modifiers. Quantitative variables such as age were presented as mean ± SD.

## Results

Sixty patients of squamous cell carcinoma (SCC) of buccal mucosa were included. There were 75% male patients (*n*=45) and 25% female patients (*n*=15). The male:female ratio was 3:1. The mean age was 56.55 years ± 5.48. Minimum age was 26 years and maximum was 66 years. According to T classification most of the cases were of T4 (>4 cm; 73.3%) (*n*=44), while T3 (>4 cm) lesions were 16.7% (*n*=10) on second number, and only 10% (*n*=6) cases were with T2-sized lesions in our case series (Table 2).

**Table 2:** Baseline characteristics

	**SCC of buccal mucosa**
	***n*=60**
Age (mean ± sd)	56.55 ± 5.48
*Gender, n (%)*	
Male	45 (75)
Female	15 (25)
*Smoking, n (%)*	
Never	10 (16.6)
Previous	16 (26.7)
Actual	34 (56.7)
*Alcohol, n (%)*	
Never	39 (65)
≤20 g/d	13 (21.7)
21 to 40 g/d	4 (6.7)
41 to 60 g/d	2 (3.3)
61 to 80 g/d	0 (0)
Unknown	2 (3.3)
*Betel nut chewing, n (%)*	
Yes	52 (86.7)
No	8 (13.3)
*Tumor size, n (%)*	
T2	6 (10)
T3	10 (16.7)
T4	44 (73.3)

According to metastatic node, the highest incidence of histopathologically positive lymph node level was level I (that is, 66.7%, *n*=40) followed by positive levels I and II (that is, 21.7%, *n*=13), and on the third number was positive levels I, II, and III (that is, 8.3%, *n*=5) while only 3.3% (*n*=2) cases were of positive I, II, III, and IV lymph node levels. Level V was not positive in any of the case Table 3.

**Table 3:** Metastatic lymph node levels

	**Frequency**	**Percentage**
Level I	40	66.7%
Levels I, II	13	21.7%
Levels I, II, III	5	8.3%
Levels I, II, III, and IV	2	3.3%
Total	60	100%

Between T classification and metastatic node, cross-tabulation showed that the incidence of metastatic lymph node in T4 (*n*=44) was the highest, that is, level I was 100% (44/44), level II was 43.18% (19/44), level III was 15.90% (7/44), and level IV was 4.5% (2/44); level V was free of metastasis. Among T3 (*n*=10) lesions, incidence of metastasis in level I was 100% (10/10), level II was 20% (2/10), and levels III, IV, and V were free of metastasis. Among T2 (*n*=6) lesions, incidence of lymph node metastasis in level I was 100% (6/6) and all other levels of lymph nodes were found free of metastasis. So the results of our study coincide with the study conducted by Y Tzu-Chen *et al.* (Figure three).

## Discussion

Buccal carcinoma commonly presents as a slow-growing mass on the buccal mucosa. Small lesions tend to be asymptomatic and are often noted surprisingly on dental examination. Pain commonly occurs as the lesion enlarges and ulceration develops. Oral intake may worsen the pain and lead to malnutrition and dehydration. Associated symptoms include bleeding, poor denture fit, facial weakness or sensory changes, dysphagia, odynophagia, and trismus [9].

A detailed medical history is important to determine the patient’s candidacy for surgery or radiation therapy. The person often has a history of betel nut chewing, tobacco, and alcohol use. A history of previous malignancies of the upper aero digestive tract should be ascertained. The appropriate management of the neck in patients with squamous head and neck cancers is critically important because the presence of cervical metastasis is the most powerful independent indicator of locoregional recurrence and overall survival rate. Clinically undetectable nodal metastasis is the worst possible scenario for treatment failure. Incidence of neck metastasis in oral SCC is reported to be 34% to 50% [13,14].

Sixty patients with squamous cell carcinoma (SCC) fulfilling the inclusion criteria who presented in the Department of Oral & Maxillofacial Surgery in 6 months’ duration of this study were included. Forty-five (75%) patients were men; 15 (25%) patients were women. The male:female ratio was 3:1, this shows male predominance which is in agreement to earlier studies by Amador *et al.*[15]. This is probably due to the fact that in Southeast Asia, snuff dipping and other tobacco-related habits are more common among men compared to women [16]. However, gender of the patient does not significantly influence the survival rate. Among the total 60 patients reported in our study, mean age was 56.55 years. Similar results have been found by Manuel *et al.* that SCC is a disease of middle age from the third to fifth decades [4].

SCC can involve any of the oral subsites and each primary site of the tumor has its own significance regarding the behavior of the tumor and its growth pattern as well as metastasis to cervical lymph nodes. Buccal mucosa is a very common presenting site of oral SCC, the higher rates of buccal mucosa carcinoma in Pakistan are likely related to the widespread practice of betel nut chewing and snuff dipping. Betel nut, composed primarily of the fruit of the areca palm and often mixed with tobacco, is placed along the buccal mucosa to induce a feeling of euphoria. Buccal carcinoma related to betel nut chewing tends to develop at an earlier age, with most cases occurring between the ages of 40 and 70 years.

The time of presentation of the cases was very late as compared to the previous other studies and most of the cases were T4 73.3% (*n*=44), followed by T3 lesions which were 16.7% (*n*=10), and only 10% (*n*=6) cases were T2 lesions in our case series.

In cases of oral SCC, metastasis in the cervical lymph nodes may occur even in T1 or T2 cases of primary tumor [17], which is a problem when establishing a therapeutic regimen. However, a possible predicting factor has not been established. Although control of the primary tumor of the oral cavity, particularly in the early stages, is often achieved, treatment failure frequently results from recurrence in the cervical lymph nodes, even among patients who initially present with no clinical evidence of neck disease. In the present study, we found that the incidence of metastasis in lymph nodes in T4 (*n*=44) was the highest, that is level I was 100% (44/44), level II was 43.18% (19/44), level III was 15.90% (7/44), and level IV was 4.5% (2/44); level V was free of any metastatic evidence of the disease. Among T3 (*n*=10) lesions, incidence of metastasis in level I was 100% (10/10), level II was 20% (2/10), and levels III, IV, and V were free of metastasis. Among T2 (*n*=6) lesions incidence of lymph node metastasis in level I was 100% (6/6) and all other levels of lymph nodes were found free of the disease so the above said results coincides with the results of Tzu-Chen *et al.*[18].

Also the previous studies support our finding that tumor size is a predictor of lymph node metastasis though they propose that tumor thickness is a more reliable factor [19,20]. This is further explained by Di Troia [21] who points to difficulty for the tumor emboli to form in small caliber lymphatics of the superficial areas, compared with wider lymphatics of deeper tissues [20]. However, tumor thickness is a radiological or histological parameter, which cannot be assessed preoperatively by clinical examination or biopsy [21-23].

This study was very selective in the sense that we selected patients only with N1 disease. On ethical grounds, patients fulfilling the criteria for functional neck dissection were only selected. There is an important controversy in treatment of neck in cases of oral cavity carcinoma as to whether to perform radical, modified, or selective neck dissection. If selective neck dissection is to be carried out, which levels need to be removed? Radical neck dissection produced significant long-term morbidity and deformity secondary to sacrifice of the spinal accessory nerve, sternocleidomastoid muscle, internal jugular vein (particularly if bilateral), and to the large incisions, skin flaps, and extent of resection. Shoulder dysfunction, paraesthesia, and chronic neck and shoulder pain were, and still are, sequelae of the radical neck dissection.

As the world is moving towards more and more conservative and less invasive approaches in treatment of oral cavity carcinomas, supraomohyoid neck dissection with the removal of level I, II, and III lymph nodes is advocated by many surgeons. Supraomohyoid neck dissection has been quite satisfactory regarding clearance of metastatic nodes, in context to 5-year survival and recurrence rates [24]. We have found that level IV involvement in our study and we recommend removal of level IV lymph nodes along with the removal of levels I, II, and III. Many studies support that level IV must also be removed along with levels I to III. Few studies showed involvement of level IV lymph nodes <10% of the cases [6,25]. In another study, level IV involvement was found to be 7% [7]. We have found the involvement of level IV nodes in 3% of the cases, so we advocate selective neck dissection (levels I to IV) in patients presenting with buccal mucosa carcinoma with N1 neck disease.

## Conclusion

Lymphatic spread from carcinoma of the buccal mucosa is low. The most common region with cervical lymph node metastasis is levels I to III in the ipsilateral neck. Involvement of level IV is seen in only 3% of patients. A more conservative approach to the neck in patients with carcinoma of the buccal mucosa is recommended. This study provides modern treatment strategies for the management of squamous cell carcinoma (SCC) of buccal mucosa.

## Consent statement

Written informed consent was obtained from the patients for publication of this study and accompanying images. A copy of the written consent is available for review by the Editor-in-Chief of this journal.

## Competing interests

The authors declare that they have no competing interests.

## Authors' contributions

HE, RW, GZ, MAR, AME, NCG, and MR conceived the study and participated in its design and coordination. HE, RW, and MR made substantial contributions to data acquisition and conception of manuscript. FM and MAR have done statistical analysis. HE and MR drafted and designed the manuscript and contributed equally to this work. RW, AME, NCG, and MR were involved in revising the manuscript. All authors read and approved the final manuscript.

## Funding

The article processing charges are funded by the Deutsche Forschungsgemeinschaft (DFG), ‘Open Acess Publizieren’.
